# Discontinuation of long-acting reversible contraceptive methods and associated factors among reproductive-age women in Shashemene town, Oromia, Ethiopia

**DOI:** 10.3389/fgwh.2024.1269302

**Published:** 2024-05-07

**Authors:** Fikru Letose, Alemtsehay Tusa, Degemu Sahlu, Yohannis Miherite

**Affiliations:** ^1^ Amref Health Africa in Ethiopia, Gambella Field Office, Gambela, Ethiopia; ^2^ Department of Maternal and Child Health, Melk Oda G/Hospital, Shashemene, Ethiopia; ^3^ Department of Public Health, College of Health Science, Salale University, Fiche, Ethiopia

**Keywords:** discontinuation, long-acting contraceptives, contraceptive, reproductive-age women, Shashemene town

## Abstract

**Background:**

The early termination of long-acting reversible contraceptives (LARCs) raises issues for the healthcare system and has the potential to affect public health. Long-acting reversible contraception has now become more widely available and used, although a sizable percentage of women still do not use it. Therefore, this study aims to assess the factors associated with the discontinuation of the LARC method among female users of health facilities in Shashemene town in Oromia, Ethiopia.

**Methods:**

A facility-based cross-sectional study was conducted in Shashemene town involving 410 study participants from nine facilities. The study participants were selected by using a systematic sampling method. The data were collected by using structured interviewer-administered questionnaires, entered into EpiData version 4.6.0.2, and exported to SPSS version 25 for analysis. Bivariate and multivariable logistic regressions were used to examine the association between independent variables and LARC discontinuation. The results were presented using the odds ratio at 95% CI. *p* < 0.05 was used to indicate statistical significance.

**Result:**

The overall prevalence of women who discontinued the LARC method before the due date was 57.2%. Having an occupation as a housewife, desire for pregnancy, unwarned side effects, effectiveness, and dissatisfaction with the service provided were the factors positively associated with the discontinuation of the contraception.

**Conclusion:**

The prevalence of the discontinuation of LARCs was high. Pre-insertion, effective counseling about the benefits, follow-up care, management of side effects, and client reassurance are recommended.

## Introduction

An unintended pregnancy is defined as a pregnancy that occurs when no children or no additional children are desired (unwanted) or earlier than desired (mistimed) (1). It is associated with an increased risk of problems for the mother and the baby. According to the World Health Organization (WHO), 74 million women in low- and middle-income countries (LMIC) experience unintended pregnancies each year. This results in 47,000 maternal fatalities and 25 million unsafe abortions each year, leading to 25 million unsafe abortions and 47,000 maternal deaths every year (2).

One of the most affordable health promotions to lower mother and child mortality by approximately 30% and 10%, respectively, is the use of modern contraceptive techniques (3). Therefore, ensuring that every pregnancy is intended and every birth is safe can prevent the vast majority of maternal and infant deaths (4). Long-acting reversible contraceptives (LARCs) are a type of modern birth control method that prevents unintended conception for an extended period without requiring additional effort from the client for at least 3 years of continuous use (5). Intrauterine copper devices (IUCDs), levonorgestrel (LNG)-releasing intrauterine system, and LNG-releasing and etonogestrel (ENG)-releasing subdermal implants are categorized as LARC methods (6–8). LARC discontinuation refers to the removal of the device used, ceasing its usage, or switching to other methods for any reason before completion of the duration (1).

Despite the improvement in the availability and utilization of long-acting reversible contraception, early discontinuation has become a major problem (9). In low-income countries (LICs), a systematic review reported a 20% discontinuation rate of LARCs within 1 year of insertion (10). In a study conducted on 21 LICs, LARCs comprised only 10% or less, of which 9% of implant users and 15% of IUCD users discontinued the method within the first year of use (11). In addition, 13.1, 26.3, and 36.7% of IUD users discontinued at the first 12, 24, and 36 months, respectively (12). The Ethiopian Demographic Health Survey (EDHS) revealed that the overall discontinuation rate of contraceptives at 12 months was 35%, of which 13% were IUD users and 11% were implant users (13). Other studies conducted in Ethiopia reported discontinuation rates of LARC use at 20% (14), 36.94% (15), 50% (16), and 66.3% (17).

Contraceptive discontinuation may be associated with external factors and individual and/or partner factors (18–20). Studies suggest that the quality of family planning services and obstetric factors such as past abortion history, number of living children, desire for more children, preferred family size, sex preference, irregular vaginal bleeding, lower abdominal pain, and abnormal vaginal discharge were linked to LARC discontinuation (14, 21–26). Sociodemographic variables, such as age, marital status, educational status, and religious concern, were found to influence LARC discontinuation. Regarding method-related variables, studies revealed that side effects (11, 14), counseling, satisfaction with the service given, and information about family planning were also identified as factors associated with LARC discontinuation (17, 21–23).

In Ethiopia, providing LARCs is a free and highly effective method for preventing unintended pregnancies (27). A woman who discontinues LARCs after 3 months faces an increased risk of unintended pregnancy by 11.6–42.3% (11). LARC discontinuation can lead to an increased fertility rate, unintended pregnancy, and associated complications (11, 28). The high LARC discontinuation rate coupled with low uptake results in a significant challenge to achieving the targeted contraceptive prevalence in Ethiopia (29).

Depending on cultural differences and time elapse, the causes for LARC discontinuation may be extremely contextual. Additionally, reducing the discontinuance of LARCs is a great way to prevent or at least minimize unintended pregnancies (30). In countries like Ethiopia, where there is high fertility and an unmet need for family planning (13), it is important to understand how long women continue to use LARCs and what factors associated with its discontinuation help improve the reproductive health of women. However, there is limited information regarding the methods of discontinuing LARCs and associated factors in the study area. Therefore, the main aim of this study was to determine the discontinuation of LARCs and its associated factors among reproductive-age women in Shashemene town, Oromia, Ethiopia.

## Method and materials

### Study area and period

The study was conducted in Shashemene town involving women aged 15–49 years. Shashemene town is the capital city of the West Arsi zone in the Oromia Region, which is 250 km far from Addis Ababa. The total town population is estimated to be 286,447, of which 49.5% are females and 34.7% are reproductive-age women. Currently, the town has two public hospitals, five health centers, two non-governmental organization (NGO) clinics, one private hospital, more than 50 private clinics, and pharmacies providing health services. Among these health facilities, all hospitals, health centers, private hospitals, NGO clinics, and nine private clinics provide LARC services. The study was conducted from May 16 to June 16, 2022.

### Study design

An institution-based cross-sectional study design was applied.

### Populations

#### Source population

All reproductive-age women who were using LARCs in the health facilities of Shashemene town.

#### Study population

All women who were current users of LARCs and visited selected health facilities of Shashemene town for contraceptive-related issues during the study period.

### Inclusion and exclusion criteria

#### Inclusion criteria

The inclusion criteria comprise all reproductive-age women who have been using LARCs for at least 12 months and visited the selected health facilities for any issues concerning the method before completion of the duration (e.g., removal, side effects, and follow-up during the actual data collection period). In addition. women who lived in the study area for at least 6 months were included in the study.

#### Exclusion criteria

The exclusion criteria comprise all women who have used LARCs outside of Shashemene town and come to the study area for the removal service. Women who visited to replace Implanon with Jadelles, Jadelles with Implanon, and implants with IUCD, or vice versa, were excluded from the study.

### Sample size determination

The required sample size was calculated by using the following population proportion formula:

$=\frac{{\left(z_{\alpha /2}\right)}^{2}\;\times \;\;P(1-P)}{d^{2}},$

where *n* is the minimum sample size; *p *= 39% prevalence of Implanon discontinuation in Ambo town (31); *z* = 1.96 (95% CI); and *d* = 0.05 margin of error. Applying the formula by substituting these values into the equation gives 373. Accordingly, 10% was added for the non-response rate, bringing the final sample size to 410.

### Sampling technique

**Figure 1:**
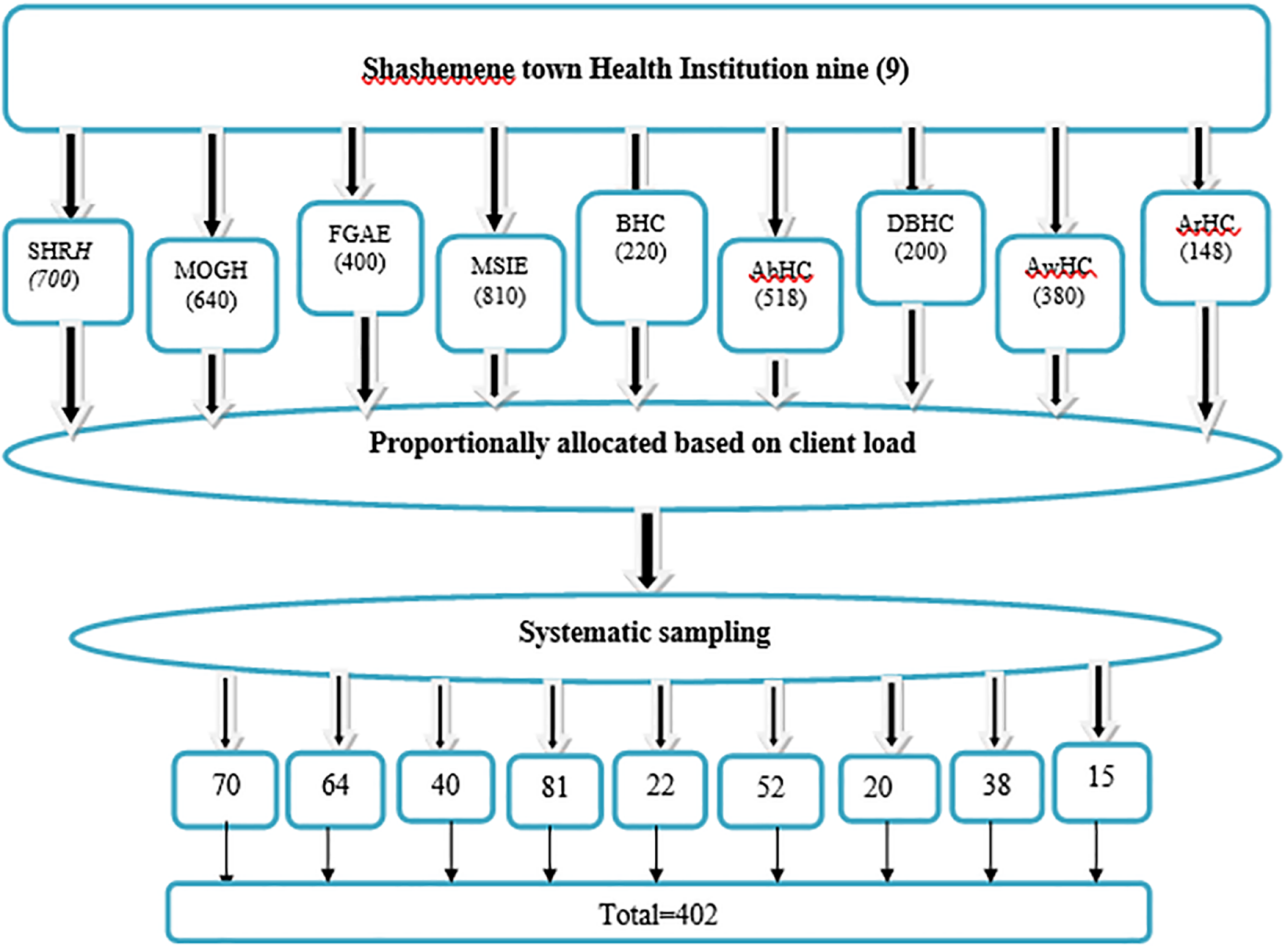
Six months’ enrolment in health institutions of Shashemene town administration in two public hospitals, five HCs, and two NGO clinics from family planning registration book for sample selection.

All public health facilities and non-governmental specialty clinics in Shashemene town, namely, the two public hospitals of Referral Hospital and Melk Oda General Hospital; five public health centers of Abosto, Awasho, Bulchana, Dida Boke, and Arada; and two non-governmental specialty clinics of Family Guidance Association and Marie Stopes International-Shashemene, which provide LARC services, were included in the study. Private health facilities were not included in this study because they were not providing full LARC services (i.e., insertion and removal). To proportionally allocate the calculated sample size, the average client flow for 6 months (i.e., from December 2021 to May 2022) at each health institution was considered before data collection. The information obtained from the 6-month enrolment record of the family planning registration book indicated that a total of 4,016 women booked for LARCs at public and non-governmental specialty clinics. The average 6-month client flow identified from the family planning registration book at Referral Hospital, Melk Oda General Hospital, Abosto HC, Awasho HC, Bulchana HC, Dida Boke HC, Arada HC, Family Guidance Association, and Marie Stopes International-Shashemene were 452, 419, 403, 410, 354, 349, 322, 618, and 689, respectively. The calculated sample size of 410 was proportionally allocated to each facility as 46, 43, 41, 42, 36, 36, 33, 63, and 70. The sampling interval “*K*” was calculated for each facility as *N*/*n*, where “*N*” stands for the number of women booked and “*n*” stands for the proportionally allocated sample. The study participants were then identified by a systematic sampling method. The data collectors approached and recruited LARC users who had come to the selected clinics for contraceptive-related reasons. The procedure was continued throughout the data collection period until the required sample size was achieved (Figure 1).

### Data collection tool and procedure

The data were collected using structured questionnaires through a face-to-face interview, which was developed based on a comprehensive review of different literature related to the topic under study. They were prepared in English and translated to Afan Oromo and Amharic for data collection. The questionnaire contains four parts: sociodemographic characteristics, contraceptive and counseling-related characteristics, health system characteristics, and obstetric-related characteristics. Two days were given to train the data collectors and the supervisor about the study objective, procedure, and research ethical rules. Informed consent was taken from the selected women, and obtaining permission for the data collection took 15–20 min.

### Study variables

The dependent variable was the discontinuation of LARCs, while the independent variables were the sociodemographic characteristics, such as age, marital status, religion, occupation, education, and family income; health system-related characteristics, such as availability of health extension workers and time taken to reach health facilities; obstetrics-related characteristics, such as parity, number of living children, history of abortion, desire for pregnancy, and time to get pregnant; contraceptive- and counseling-related characteristics; and information about contraceptive (e.g., history of family planning before use, type of contraceptive used before LARCs, responsible body for choosing LARCs, counseling, and satisfaction with service given).

### Data quality assurance

To assure the quality of data, we pre-tested the questionnaire from May 9 to 11, 2022 at 01 Kebele (Hage) Health Center found outside of the actual study area. Five percent of the calculated sample of women was enrolled to check the validity and reliability of the questionnaire. After pre-testing the questionnaire, revisions and amendments were made accordingly. Training on how to interview and check the questionnaire for completeness was given to the data collectors. The supervisors and the principal investigator checked and revised the completeness of the questionnaire and offered necessary feedback to the data collectors.

### Data management and analysis

The collected data were entered using EpiData version 4.6 and exported to SPSS 23 software for cleaning, recoding, categorizing, and analyzing. A bivariate analysis was done to see the association between independent and outcome variables. Variables with a *p*-value of 0.2 during the bivariate analysis were included in the multiple logistic regression analysis to assess the relative effect of confounding variables. The outcome variable is categorical; hence, the adjusted odds ratio (AOR) was calculated using a multiple logistic regression model. After the multivariate analysis had been done, the AOR was used to measure the strength of the association between the dependent and independent variables, while the 95% CI and *p*-value were used to assess whether the association was significant.

### Ethical consideration

An ethical assurance letter was received from SLU College of Health Sciences, Department of Public Health. It was presented to the health office of Shashemene town to ask for official permission to undertake research activities in the selected health facility. Written informed consent was obtained from each participant after the investigator had explained the nature, purpose, and procedure of the study. The signature of each participant was used on the willingness confirmation. The entire study's participants were informed that data would be kept private and confidential and used only for research purposes. The participants were also assured that they had the right to refuse or withdraw if they were not comfortable at any time.

## Results

### Sociodemographic characteristics

A total of 402 women responded to the questionnaire, forming a response rate of 98%. The mean age of the respondents was 26 years (±4.6). Approximately 241 (60.0%) were Oromo by ethnicity, and 150 (37.3%) were Muslims by religion. Most of the respondents, 268 (91.5%), were married. Two hundred fifty-three (62.9%) respondents attended secondary education. Regarding occupation, the majority of the respondents, 230 (57.2%), were housewives. More of the respondents have a monthly income of 2,000–3,000 ETB per month (Table 1).

**Table 1:** Sociodemographic characteristics of long-acting reversible contraceptive users in the health facilities of Shashemene town, Southern Ethiopia, 2022 (*n* = 402).

Variable	Frequency	Percentage
Age, years		
<20	25	6.2
20–29	226	56.2
30–39	117	29.1
40+	34	8.5
Marital status		
Married	368	91.5
Single	22	5.5
Divorced/separated	7	1.7
Widowed	5	1.2
Educational status (*n* = 402)		
No formal education	22	5.5
Primary education	253	62.9
Secondary education	91	22.6
Higher education/University/College	36	9.0
Occupational (*n* = 402)		
Housewife	230	57.2
Government employed	43	10.7
Private employed	44	10.9
Merchant	85	21.1
Family income		
<2,000	29	7.2
2,000–3,000	220	54.7
3,000+	153	38.1

### Health system-related characteristics

The majority of women, 342 (85.1%), had access to health extension workers in their catchment, and more than half of the women, 252 (62.7%), were living within 30–60 min walking distance from the health facilities.

### Obstetric-related characteristics

Among the women who ever used LARCs, only three (1%) had no history of live birth. Nine (2.2%) had no child; 122 (30.3%) had a history of abortion; and 177 (44%) desired to become pregnant (Table 2).

**Table 2:** Obstetric history of women who used LARC in 2021/2022 in Shashemene town, Oromia, South Ethiopia, 2022 (*n* = 402).

Variable	Frequency	Percentage
Parity		
0	3	0.7
1–2	137	34.1
3–4	203	50.5
5+	59	14.7
Women having living children (*n* = 402)		
0	9	2.2
1–2	139	34.6
3–4	198	49.3
5+	56	13.9
History of abortion		
Yes	122	30.3
No	280	69.7
Desire for pregnancy		
Yes	177	44.0
No	225	56.0

### Contraceptive and counseling-related characteristics

From the total number of women interviewed, approximately three-fourths (74.4%) used other contraceptive methods before LARCs. For instance, of these women, approximately 150 (50.3%) had been using injectables. Regarding method selection, nearly 2/3 (64.7%) of the women decided to use LARCs by their own choice. Among the types of LARCs, 220 (54.7%) women used Implanon. Among the total study participants, the number of women who were counseled about the benefits, side effects, and effectiveness of LARCs was 199 (49.5%), 203 (50.5%), and 227 (56.5%), respectively. One hundred ninety-five (48.5%) of the respondents mentioned having been satisfied with the service given before the insertion (Table 3).

**Table 3:** Contraceptive and counseling-related characteristics of women who used LARCs in 2021/2022 in Shashemene town, Oromia, Southern Ethiopia, 2022 (*n* = 402).

Variable	Frequency	Percentage
Contraceptive use before LARCs		
Yes	299	74.4
No	103	25.6
Type of contraceptive used before LARCs		
Injection	150	50.2
OCP	93	31.1
OCP and injection	45	15.2
Others	10	3.5
Method selection		
Myself	260	64.7
My husband	12	3.0
Health professional	83	20.6
Others (health extension workers, neighbors, etc.)	47	11.7
Type of LARC used		
Implanon	220	54.7
Jadelle	110	27.4
IUD	72	17.9
Counseled about the benefits of LARC		
Yes	199	49.5
No	203	50.5
Counseled about the side effects		
Yes	203	50.5
No	199	49.5
Counseled about effectiveness		
Yes	227	56.5
No	175	43.5
Information participants got during counseling (satisfied)		
Yes	216	53.7
No	186	46.3

### Magnitude of long-acting reversible contraceptive discontinuation

**Figure 2:**
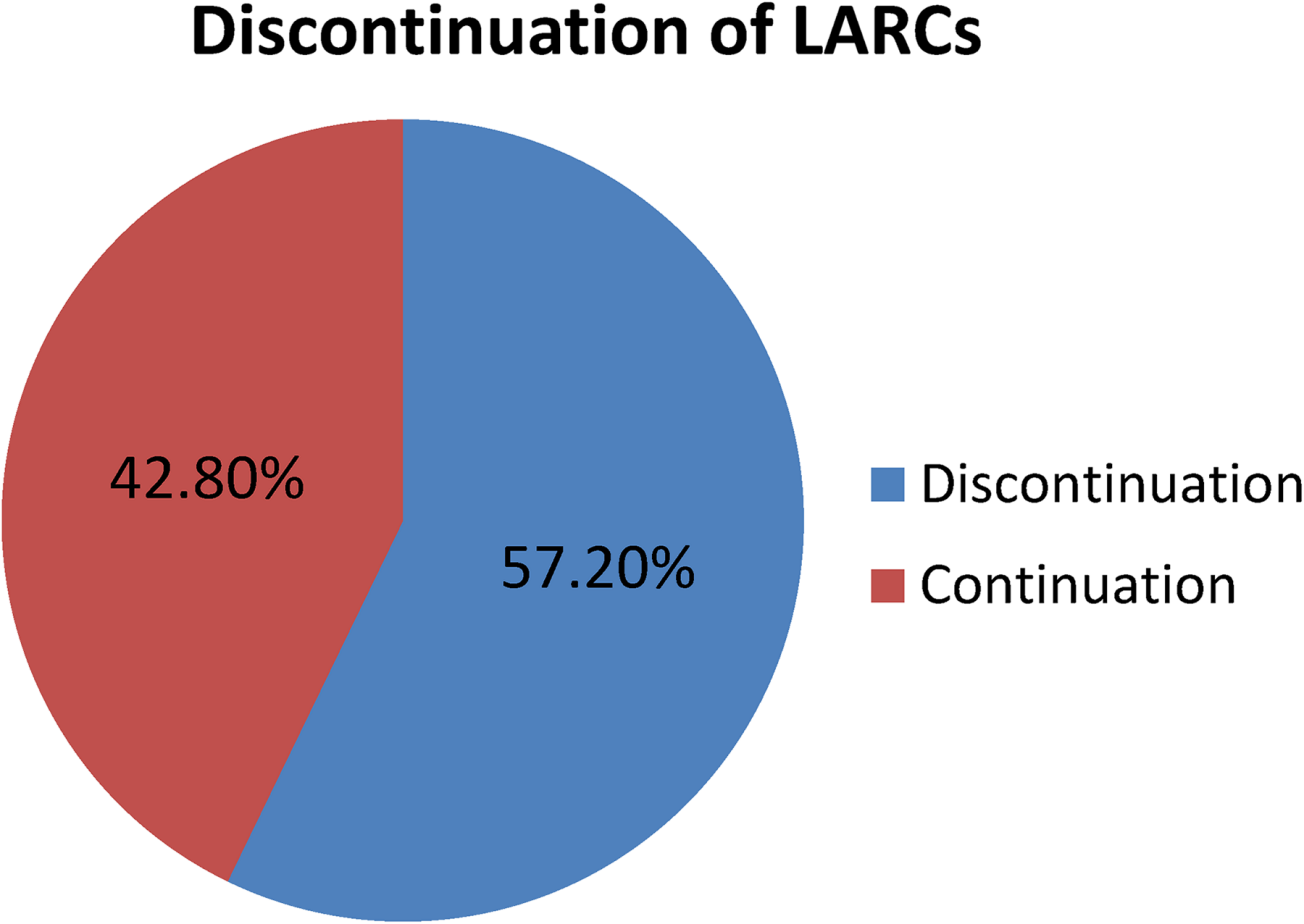
Proportion of the LARC discontinuation rate among LARC users in Shashemene town, Southern Ethiopia in 2022.

**Figure 3:**
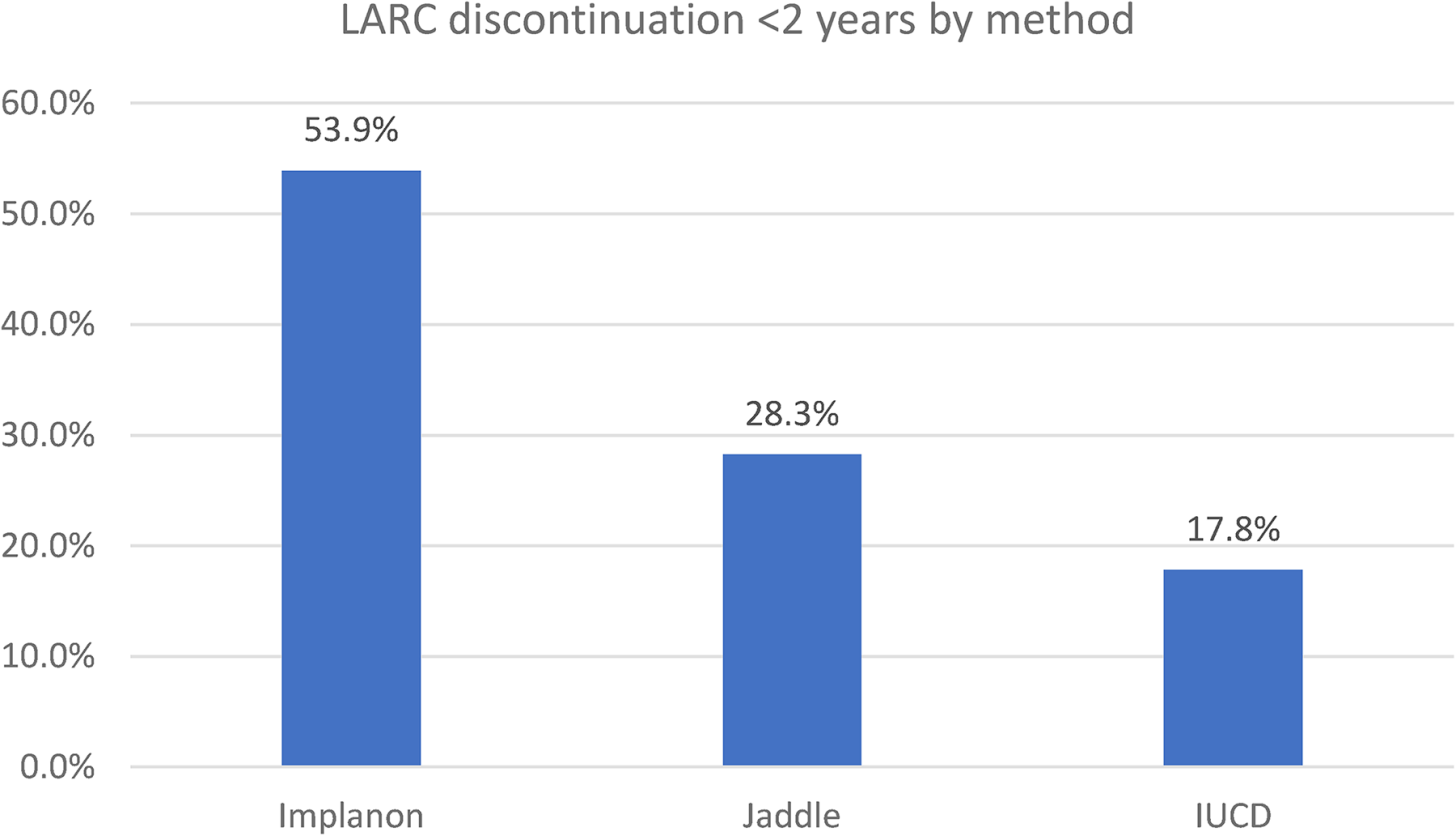
LARC discontinuation of <2 years by method.

**Figure 4:**
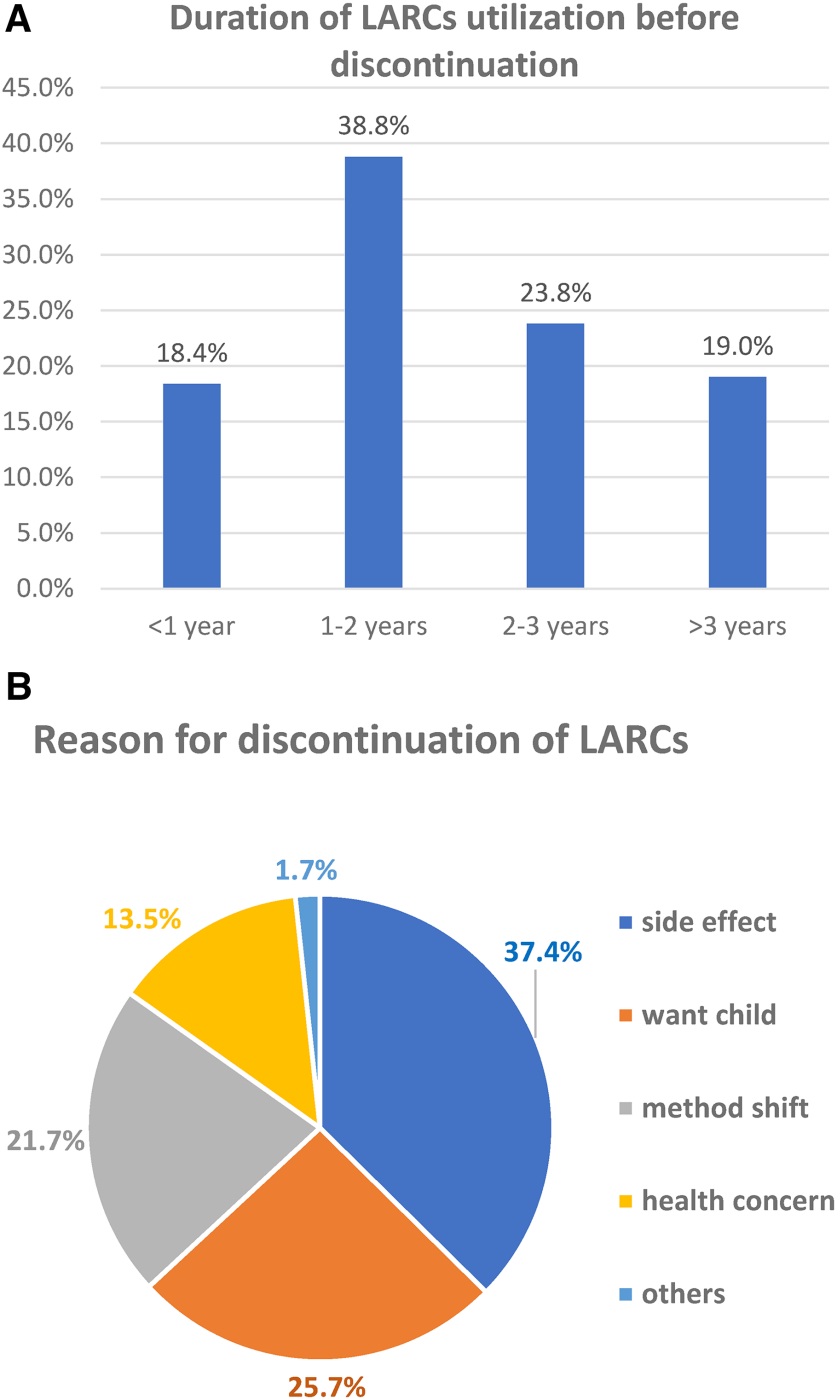
(**A**) Duration of LARC utilization before discontinuation. (**B**) Reason for discontinuation among women who ever used LARC in 2021/2022 in Shashemene town, Southern Ethiopia (*n* = 230).

The study revealed that, among the study participants who ever used LARCs, 18.4%, 57.2%, and 81% of women discontinued the service at 12, 24, and 36 months, respectively. Moreover, 38.8% and 23.8% of women discontinued it at 12–24 and 24–36 months, respectively. Among the study participants, only 76 (19%) of women used LARCs for at least 36 months. Women who removed LARCs at the due date comprised 7.5%. Regarding the types of LARCs discontinued at 12 months, the proportion of women who discontinued Implanon, Jadelle, and IUCD was 51.4%, 31.1%, and 17.6%, respectively (Figures 2–4).

### Reasons for discontinuation of LARCs

**Figure 5:**
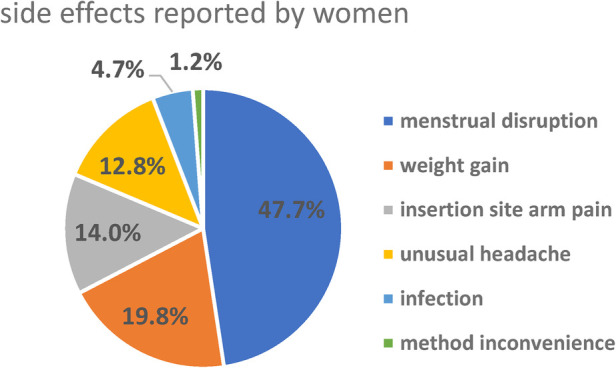
Side effects reported by women.

More than one-third (37.4%) of women discontinued LARCs within 3 years of utilization mainly due to emerging side effects. This was followed by 84 (25.7%) who desired to have a child (Figure 5). The most common complaints among women who developed side effects were menstrual disruption (58, 47.7%), weight gain (24, 19.8%), insertion site arm pain (17, 14.0%), unusual headache (16, 12.8%), infection (6, 4.7%), and method inconvenience (1, 1.2%) (Figure 5).

### Factors associated with discontinuation of reversible long-acting contraceptives

From the bivariate logistic regression analysis, factors such as age, educational status, occupational status, desire for pregnancy, counseling about the benefit of LARCs, counseling on the effectiveness of LARCs, and satisfaction with the service given have a *p*-value of ≤0.2 and are considered for multivariable logistic regression.

Factors from the bivariate logistic regression analysis with a *p*-value of ≤0.2 were entered into the multivariable logistic regression analysis. With the multivariable logistic regression analysis, considering a *p*-value of ≤0.05, the independent predictors of LARC discontinuation were maternal occupation, desire for pregnancy shortly, lack of counseling about the side effects of LARCs, no counseling on the effectiveness of LARCs, and not being satisfied with LARC service provision (Table 4).

**Table 4:** Bivariate and multivariate analysis of the factors associated with the discontinuation of long-acting reversible contraceptives in Shashemene town Southern Ethiopia, 2022 (*n* = 402).

Variables	Discontinuation of LARCs		COR (95% CI)	*p*-value	AOR (95% CI)	*p*-value
	Yes (%)	No (%)				
Age						
<20	13 (52.0%)	12 (48.0%)	1			
20–29	162 (71.7%)	64 (28.3%)	0.43 (0.18–0.98)	0.047	0.59 (0.17–1.98)	0.394
30–39	41 (35.0%)	76 (65.0%)	2.00 (0.84–4.80)	0.117	2.34 (0.68–8.02)	0.175
40+	14 (41.2%)	20 (58.8%)	1.54 (0.54–4.38)	0.411	5.53 (0.26–24.16)	0.073
Educational status						
No formal education	10 (45.5%)	12 (54.5%)	1		1	
Primary education	191 (75.5%)	62 (24.5%)	0.27 (0.11–0.65)	0.004	0.47 (0.14–1.56)	0.220
Secondary education	24 (26.4%)	67 (73.6%)	2.33 (0.89–6.08)	0.085	4.82 (0.24–18.67)	0.093
Higher education	5 (13.9%	31 (86.1%)	5.17 (1.46–18.27)	0.011	8.24 (0.41–47.96)	0.099
Occupational status						
Housewife	184 (80.0%)	46 (20.0%)	0.09 (0.05–0.17)	0.000	0.09 (0.04–0.17)*	*0*.*000*
Government employee	4 (9.3%)	39 (90.7%)	3.83 (1.23–11.90)	0.020	0.69 (0.15–3.09)	0.630
Private employee	18 (40.9%)	26 (59.1%)	0.56 (0.26–1.22)	0.147	0.45 (0.16–1.27)	0.135
Merchant	24 (28.2%)	61 (71.8%)	1		1	
Desire for pregnancy						
Yes	104 (46.2%)	121 (53.8%)	2.87 (1.89–4.36)	0.000	3.69 (1.90–7.14)*	<0.001
No	126 (71.2%)	51 (28.8%)	1		1	
Counseled about the benefits of LARC						
Yes	74 (37.2%)	125 (62.8%)	5.60 (3.63 –8.66)	0.000	0.79 (0.29–2.12)	0.643
No	156 (76.8%)	47 (23.2%)	1		1	
Counseled about the side effects of LARC						
Yes	152 (76.4%)	47 (23.6%)	1		1	
No	78 (38.4%)	125 (61.6%)	5.18 (3.36 –7.98)	0.000	2.57 (1.10–6.01)*	0.029
Counseled about the effectiveness of LARC						
Yes	122 (69.7%)	53 (30.3%)	1		1	
No	108 (47.6%)	119 (52.4%)	2.53 (1.67–3.83)	0.000	2.77 (1.43–5.39)*	0.003
Satisfied with the service given						
Yes	162 (78.3%)	127 (65.1%)			1	
No	68 (34.9%)	45 (21.7%)	6.72 (4.31–10.46)	0.000	2.83 (1.15–6.95)*	0.023

OR, odds ratio; AOR, adjusted odds ratio; LARCs, long-acting reversible contraceptives.

* *p* < 0.05.

Among the sociodemographic variables, the occupational status of women was found to be associated with the discontinuation of LARCs. Women having the occupation of housewives were approximately 90% less likely to discontinue LARCs compared to their merchant counterparts (AOR = 0.09; 95% CI: 0.04, 0.17). The odds of discontinuing LARCs among women who desire to be pregnant were 3.7 times higher than those of their counterparts (AOR = 3.7; 95% CI: 1.90, 7.14). Regarding the counseling status of women as regards the side effects of LARCs, those who did not obtain counseling were 2.6 times more likely to discontinue LARCs compared to their counterparts (AOR = 2.6; 95% CI: 1.10, 6.01). Women who were not counseled about the effectiveness of LARCs were 2.8 times more likely to discontinue LARCs compared to their counterparts (AOR = 2.8; 95% CI: 1.43, 5.39). Women who were not satisfied with the service given were 2.8 times more likely to discontinue LARCs compared to their counterparts (AOR = 2.8; 95% CI: 1.15, 6.95) (Table 4).

## Discussions

Nevertheless, 30% of maternal deaths and 10% of infant deaths were reduced by LARCs, which is one of the most practical and cost-effective methods, by ensuring that every pregnancy is desired, and every birth is safe (3, 4). The overall prevalence of LARCs was found to be 18.4%. The current study finding was consistent with the studies conducted at Hawassa City in Southern Ethiopia at 17.7% (29), Bahir Dar City in Northwest Ethiopia at 17.2% (32), and slightly less than the urban public health facilities of Ethiopia (32).

The current study's percentage of LARC discontinuation at 36 months was lower than the 65% found in a study conducted in Debre Tabor, Northwest Ethiopia. This variation could be a result of the subjects or the design of the study. The participants from Debre Tabor, in contrast to the current study, included both urban and rural populations. Urban inhabitants might be more aware of the consequences of contraception. Additionally, efforts may have been taken to enhance counseling, particularly for the women in the current study who had issues with weight gain and menstruation disruption. The result in the current study was also lower than those in studies conducted in Ambo (38.2%) (26) and Arsi zone (25%) (19). This discrepancy might be caused by differences in the study design, sample size, study duration, lack of pre-insertion counseling, satisfaction with the given service, and sociocultural differences of respondents across the study areas.

Among the types of LARCs discontinued in this study, the prevalence of Implanon (41.8%) and IUCD (15.2%) was found to be less than the secondary data analyzed from the EDHS of 61 and 45% by 36 months, respectively (12). This might be caused by the discrepancy in the population structure of the national pattern and variation in the duration of utilization before discontinuation.

The study revealed that the main reasons for the discontinuation of LARCs were the emerging side effects (37.4%), desire to have a child (25.7%), method shift from LARCs to short-acting reversible contraceptive methods (21.7%), and health concern (13.5%). Menstrual disruption (47.7%) was the main side effect faced by discontinuers. Menstrual irregularity may not cause serious health problems but interferes with daily activity, especially sexual activity with their husband. Discontinuation due to side effects was a result of inadequate counseling and information on the possible side effects before and following the insertion of LARCs. Giving adequate counseling and information on the side effects and effectiveness of LARCs likely improves service satisfaction and ensures the continuation of LARCs (13, 16, 29).

Among the independent predictors, the occupational status of women was found to be associated with the discontinuation of LARCs. Women who were housewives were approximately 90% less likely to discontinue LARCs compared to their merchant counterparts. This may be because women having the occupation of merchant might be more likely to be educated and have access to information about modern family planning from different sources.

The current study also revealed that the odds of discontinuing LARC among women who desired to be pregnant shortly were 3.6 times higher than their counterparts. This is consistent with studies conducted in Kenya (33), Bahir Dar City (34), Debre Markos town (35), and Hawassa City (29). This might be because the culture of the community influences married women to bring a child to ensure the ability of the women to bear a child. In addition to this, 62.4% of women in the present study were aged 18–29 years. The majority of the study participants were young; hence, they might intend to have more children and discontinue the contraceptives.

The present study showed that women who were not counseled about the side effects of the LARCs were 2.6 times more likely to discontinue LARCs compared to their counterparts, which is consistent with the study done in Ethiopia through a systematic review (13) of the Hawassa (29) and Kersa districts (36). The current study also revealed that counseling women about the effectiveness of LARCs was negatively associated with LARC discontinuation. The most cited reason for this effect was that effective counseling at the insertion time on the possible side effects of the contraceptive will help the mother accept its possible side effects and minimize its early removal. In addition to this, the use of an appropriate counseling type and timing might also help women cope with minor side effects and strengthen the continuation of the method.

According to the study, women who were unsatisfied were 2.8 times more likely to stop using LARCs than those who were satisfied. This might be the result of incorrect pre-insertion counseling and a lack of seclusion, which led to frustration and quitting. This finding is consistent with that of the study conducted in Makelle City, Dale district, Kambata zone in Southern Ethiopia, and Vietnam (18, 21, 27, 36). This might be due to women's lack of interest in the method chosen. Furthermore, the confidentiality of the service, elucidation of the service supplier, communication skills, and other service provisions during the insertion of LARCs may contribute to LARC discontinuation. Therefore, providing quality reproductive health services to clients can lead to better satisfaction and use of the services.

### Limitation

The study's cross-sectional design prevented it from determining the cause-and-effect link. In addition, the possibility of recall bias could not be ruled out, and to study determinant factors, a cross-sectional design study has limitations.

### Conclusion

In this study, the overall discontinuation of LARCs among women was high (57.2%). The independent predictors of LARC discontinuation among women were maternal occupation as a housewife, desire for pregnancy, not counseling on side effects, not counseling on the effectiveness of LARCs, and not being satisfied with the service given.

Addressing low community awareness through community-level awareness creation programs and health education ensures long-acting reversible contraceptive use. Providing pre-insertion counseling on side effects, effectiveness, and benefits is also beneficial. Early management and reassurance can decrease discontinuation and enhance retention. A further comprehensive study of this area that includes health professionals and is community-based is recommended.

## Data Availability

The original contributions presented in the study are included in the article/Supplementary Material, Further inquiries can be directed to the corresponding author.
